# Genomic intensive care: should we perform genome testing in critically ill newborns?

**DOI:** 10.1136/archdischild-2015-308568

**Published:** 2015-09-14

**Authors:** Dominic JC Wilkinson, Christopher Barnett, Julian Savulescu, Ainsley J Newson

**Affiliations:** 1 Faculty of Philosophy, Oxford Uehiro Centre for Practical Ethics, University of Oxford, Oxford, UK; 2 Robinson Institute, Discipline of Obstetrics and Gynaecology, University of Adelaide, Adelaide, South Australia, Australia; 3 Newborn care unit, John Radcliffe Hospital, Oxford, UK; 4 SA Clinical Genetics Service, Women's and Children's Hospital/SA Pathology, North Adelaide, South Australia, Australia; 5 School of Paediatrics and Reproductive Health, University of Adelaide, Adelaide, South Australia, Australia; 6 Centre for Values, Ethics and the Law in Medicine, School of Public Health, University of Sydney, Sydney, New South Wales, Australia

**Keywords:** Ethics, Genetics, Neonatology, Intensive Care

In newborn intensive care units (NICUs), the science and art of prognostication often have life and death implications. Approximately 5% of infants admitted to NICU die.^1^ The majority of deaths are preceded by decisions to withdraw or withhold life-sustaining treatment,^1^ following discussions between the family and clinical team. These decisions are based on an assessment of an infant's chance of survival and on the predicted duration and nature of the infant's survival if treatment is provided.^2^

A variety of clinical, biochemical, genetic and radiological tests have traditionally been employed to estimate prognosis in the NICU. While chromosomal microarray is now commonly used for critically ill neonates with congenital malformations, new forms of genetic and genomic testing^3^ have started to become available in intensive care.^4^ They could aid critical care decision-making by predicting functional outcome, important comorbidities^5^ or poor prognosis despite treatment^4^ (box 1).

Box 1 **Genomic testing and ethical dilemmas: hypothetical case studies**

1. A term newborn infant is born in poor condition in the setting of meconium-stained liquor and variable decelerations. The infant requires resuscitation, including intubation and is transferred to the neonatal intensive care unit. The infant has early evidence of encephalopathy with refractory seizures. Should the infant have rapid whole genome or exome sequencing to look for a possible underlying inborn error of metabolism or epileptic encephalopathy?^4^
2. A newborn infant is born extremely preterm at 24 weeks’ gestation. At 1 week of age, the infant remains critically unwell, and has developed evidence of sepsis and necrotising enterocolitis. Chromosomal microarray testing had been performed on cord blood, and now reveals a microdeletion that has been associated with an increased risk of autism and schizophrenia. Should this information be revealed to the infant's parents? Should it be used in decision-making about continuation of intensive care and surgery for the infant's necrotising enterocolitis?
3. A newborn infant was diagnosed with complex congenital heart disease antenatally. The infant's parents declined amniocentesis following the anomaly scan as it would not have changed their decision to continue the pregnancy. Postnatally, the infant is confirmed to have hypoplastic left heart syndrome. The infant has minor dysmorphic features. The infant's parents are requesting full active treatment, and local practice includes the option of cardiac transplantation. Should the infant have genomic testing prior to listing for transplantation? What features on testing would make the infant ineligible for transplantation?

## Genomic testing

Genetic testing is already available, and is widely used in newborn intensive care. This includes conventional karyotyping, fluorescent in-situ hybridisation, array comparative genomic hybridisation (aCGH) and sequencing of a gene or genes using Sanger (‘traditional’) DNA sequencing in situations where a single gene disorder is suspected. More recently, multiple gene sequencing panels using next-generation sequencing (NGS) techniques, whole exome sequencing (WES; sequencing of the coding/exonic regions of the genome) and whole genome sequencing (WGS) have become increasingly available in the clinical setting.^3^ ^6^ These newer methods differ in both the potential depth and breadth of analysis and information obtained.

Until recently, WES and WGS have largely been used in research. However, the rapidly falling cost and increasing speed of NGS have made clinical use possible. Some centres are gaining experience in the use of WES and WGS in selected patients with difficult-to-diagnose illness.^5^ ^7^

Genomic testing capable of diagnosing hundreds of genetic conditions in ‘one test’ might have particular application in the NICU since this is the time point when many congenital and genetic disorders become apparent. The full phenotype of the illness may not yet be apparent, meaning that diagnosis by conventional means is often delayed. In one recent series, WGS with targeted analysis was completed in as little as 50 h, yielding a genetic diagnosis in 20 out of 35 infants thought to have a likely monogenic disorder tested prospectively.^8^ The authors described a newborn infant with refractory seizures, from whom life support was withdrawn after 5 weeks of intensive care and multiple investigations and trials of therapy. WGS revealed a homozygous gene mutation previously described in a lethal neonatal seizure disorder.^4^ ^8^

### Different uses of genomic testing

Genomic testing (ie, NGS panels, WES, WGS) could theoretically be used at multiple different time points, for example, preconception, preimplantation, antenatally or postnatally. Here, we focus on the use of genomic testing after birth in critically ill infants.

Genomic testing may be used for diagnosis. It may also be used prognostically for critically ill infants in several different, though overlapping, ways. Genomic testing might be used for *treatment modification* to identify specific pharmacological or other treatments that are likely or unlikely to be of benefit given an infant's outcome. Even in the absence of specific treatment, it could be used for *anticipation or information*, providing parents with advance knowledge of potential future problems as well as the risk of recurrence in future offspring. Genomic testing might also be used to help inform *treatment limitation*, either because it is relevant to the best interests of the infant, to parental preferences for treatment or to resource allocation.

There will be different levels or degrees of influence of genomic testing over the above categories of clinical decisions. In some situations, information from genomic testing may make only a small contribution to parents’ and doctors’ decisions about treatment. Often, regardless of whether there is a genomic test result that indicates a problem, there is sufficient uncertainty about the benefits of continuing treatment (or a sufficiently worrying prognosis) that treatment limitation would be reasonable. But in other situations, genomic testing could have more influence. Treatment would continue in the absence of a specific test result, yet in its presence, treatment limitation will be seriously considered.

## The ethics of genomic testing for critically ill infants

The over-arching ethical considerations around genomic testing for critically ill infants in the NICU are summarised in table 1. These echo those that have been extensively discussed around genomic testing elsewhere.^9^ ^10^

**Table 1:** A summary of general ethical considerations for and against genomic testing in the NICU

In favour of genomic testing in the NICU	Points of concern with genomic testing in the NICU
Actionability Genomic testing may yield information that is relevant for medical decision-making in the NICU A test is generally thought to be of value if it generates information that has both clinical utility and validity; providing actionable information^11^ However, it will often be difficult to predict whether genomic testing will lead to actionable information (particularly while genomic knowledge is at an early stage), and it may do so only in a minority of cases Cost-effectiveness Genomic testing may save costs by preventing the need for expensive and prolonged diagnostic testing (including traditional Sanger sequencing), as well as by avoiding prolonged, expensive and non-beneficial intensive care treatment.^4^ The costs of genomic tests have fallen rapidly *However, the after-sequencing costs, including bioinformatics and interpretation may be substantial*^12^ Access to testing Genome testing will soon be available to parents prenatally or postnatally from private companies. It would potentially be better to provide information within a healthcare system with access to support and interpretation. It might also be unfair to deny parents (who cannot afford it) the option of testing that might be relevant to clinical management decisions Consistency The principles of use of genomic testing in the NICU are similar to more conventional investigations that are widely used, such as karyotype, single-gene sequencing, MRI or metabolic tests	False-positive results Genomic testing may lead to incorrect diagnoses. Even very accurate tests with high sensitivity and specificity yield significant numbers of false positives when applied to a very large number of genes.^13^ False-positive results may also arise because variations within or adjacent to disease-causing genes may be predicted by commonly used software packages (PolyPhen, Mutation Taster) to be pathogenic, but in the fullness of time are proven not to be. A significant proportion of mutations that were previously identified in published literature as causative of disease are now believed to be incorrect^14^ Incidental findings Testing could reveal genetic mutations that confer risk of adult-onset illnesses such as cancer or neurodegeneration.^13^ ^15^ ^16^ Release of this information may have no bearing on current clinical decision-making, prevents children deciding whether to undergo testing in the future (removing their right to an ‘open future’) and could lead to psychological harm in the family^17^ *However, diagnosis of these conditions could be beneficial for the child psychologically and in terms of the child's autonomy (eg, by helping the child make decisions about education and career),*^18^ *and could have major implications for the health of the child's parents.*^16^ *See also ‘genetic privacy’ below* Uncertain results Genome testing may identify variants of uncertain significance (VUS).^19^ A VUS is a DNA sequence alteration or copy number variation (deletion/duplication), which is not common in the general population, but for which a definite link to human disease cannot be made on current data. Some VUS’ will be redefined as pathogenic mutations in the near future as more cases are ascertained; others will be classified as non-pathogenic with time. For this reason, post-test interpretation can be challenging, highly complex, non-definitive and potentially confusing for parents Genetic privacy Testing could impact on future employment or ability to access insurance. Identification of genetic abnormalities in the child may also lead, by inference or further testing, to identification of mutations in the parents and extended family *However, these concerns apply equally to genetic and other tests already in use and accepted in the NICU. Notions of ‘privacy’ with respect to children are also difficult to uphold. In some jurisdictions, these concerns may be partly addressed by existing legislation or regulation*^19^

This table necessarily condenses a complex debate, which is discussed in depth elsewhere.^6^ ^7^ ^9^ ^10^

Opposing considerations are shown in *italics*.

NICU, newborn intensive care unit.

The benefits and risks of such testing in critically ill infants could lead to several different strategies. Faced with the challenges of interpretation of genomic information and the implementation of testing in critically ill infants, one option would be to refrain from using newer forms of genomic tests in the NICU, at least until their clinical utility and validity are better demonstrated. However, in the NICU, it appears that genomic testing will at least sometimes be justifiable. In case 1 (box 1), for example, identification of a mutation in STXBP1 would lead to diagnosis of Ohtahara syndrome, for which, folinic acid therapy may be helpful.^20^ In the recent case series, WGS had a much higher rate of genetic diagnosis than conventional testing (20/35 infants vs 2/35 infants), and led to a substantial favourable impact on acute clinical management in 4 out of 20 infants.^8^ For example, in one infant with refractory hypoglycaemia, identification of a gene for focal familial hyperinsulinism led to modification of planned surgery with subsequent resolution of symptoms.^8^ Genomic testing could equally lead to avoidance of non-beneficial treatment. Six of the 20 infants receiving a genetic diagnosis in the WGS study were subsequently shifted to palliative care. A genomics clinic in Wisconsin has described a critically ill infant with severe cryptogenic liver disease in whom WGS identified a mutation in the *C10orf2* gene, previously associated with progressive neurological deterioration, which contributed to a decision not to proceed with liver transplantation.^5^

Further, these sequencing techniques are highly likely to move into clinical practice over the next few years to complement or replace existing tests. aCGH has already replaced karyotyping as a first-line investigation for congenital abnormalities,^21^ and raises similar issues to WES and WGS (eg, case 2), although on a more contained scale.^10^ Even if unavailable through a public healthcare system, genomic testing may be sought privately, for example, through sequencing of fetal DNA in maternal plasma.^22^

Finally and importantly, although the scale of the problems may be greater, the ethical issues raised by the newer technologies (WES/WGS) are similar in principle to the ethical issues raised by other genetic tests and indeed other diagnostic and prognostic tests in the NICU.

The key question, we suggest, is, therefore, not *whether* genomic testing is used, but *when* and *how* it is used.

### When should genomic testing be used in the NICU?

#### Targeted diagnostic testing

The most obvious and least controversial policy for implementation of genomic testing in the medium term is its use in a targeted fashion for diagnosis (eg, case 1, box 1).^10^ This would involve careful selection of patients for whom genomic testing is most likely to identify a pathogenic mutation. Examples include infants with a suspected, but undiagnosed, metabolic condition where genomic testing could provide diagnostic information, not available from biochemical screening. There are numerous conditions that are not included on newborn biochemical screening tests that would be diagnosed on genome testing, including mucopolysaccharidoses (Hunter syndrome, Hurler syndrome), congenital disorders of glycosylation and disorders of cholesterol metabolism (Smith–Lemli–Opitz syndrome).

Genomic testing might be used in place of repeated Sanger gene sequencing for infants with dysmorphism, complex neuromuscular presentations (eg, arthrogryposis), or congenital abnormalities that could be related to multiple different genes. This could increase the diagnostic yield in this group of infants.^8^

#### Targeted prognostic testing

Genomic testing could (and arguably should) also be used to provide additional prognostic information that would be potentially actionable. This includes the examples of cases 2 and 3 (box 1). While the general presumption is not to test young children for adult-onset conditions, the setting of critical illness in the NICU means that a different approach may be warranted. Future health states, even if not treatable or preventable (and perhaps especially if they are not treatable or preventable) may be relevant.

There are two arguments in favour of a more permissive approach to testing here. The first is related to the ethical uncertainty inherent in the NICU.^2^ One way of understanding the best interests approach to treatment decisions in the NICU is as an attempt to weigh up the benefits against the burdens of providing treatment. For infants with very severe congenital malformations, hypoxic brain injury or extremely premature infants at the borderline of viability, there is a sufficiently fine balance between benefits and burdens that some families will choose treatment continuation, while others choose treatment withdrawal. There can often be no ethical presumption one way or the other: both are reasonable choices to make. Additional burdens in the future life of an infant with seriously compromised health (eg, an additional risk of mental illness, cognitive impairment or early onset malignancy) might be judged by some parents to tip the balance in favour of not continuing treatment. Conversely, the absence of additional burdens might lead parents to request continued treatment.

The second reason in favour of testing is that these situations may be judged to fall into a ‘zone of parental discretion’.^2^ ^23^ Information about an infant's long-term prognosis may be relevant both to parents’ own interests and to their evaluation of the interests of the child. Of course, genomic testing may not yield information that parents judge relevant to their own or their child's interests. It may even yield unhelpful information about possible future risks with wide CIs. However, this does not mean that there should be a presumption against testing. Rather, following appropriate counselling and discussion, parents’ considered views and values should be given serious consideration in jointly reaching a decision.

In cases like case 2 and case 3, a broader range of information should be sought than in diagnostic testing. Case 2 and case 3 are indicative of those infants where there is a genuine question about whether or not to provide treatment. Prognostic testing may, therefore, be appropriate here as specific test results may be important and relevant to parents and treating team. In contrast, prognostic testing should not routinely be conducted for infants where there is no pre-existing question about provision of life-sustaining treatment. For example, given the uncertainties about results and the ethical considerations summarised in table 1, it would not be appropriate to embark on prognostic genomic testing for moderately or mildly premature infants, or infants with readily treatable congenital abnormalities. (Diagnostic testing in the latter cases may still be relevant if there is suspicion of an underlying syndrome).

One further interesting question, beyond the scope of this paper, is whether in cases like case 3, testing may proceed even in the absence of parental consent. Here, prognostic information would be highly relevant to decisions about treatment on the basis of the child's best interests or on the basis of resource allocation.

### How should genomic testing be used in the NICU?

There are several key strategies for addressing the ethical challenges arising from the use of genomic testing in critically ill infants.

First, it will clearly be important for parents to have an opportunity to make an informed and considered decision before agreeing to genomic testing. Some parents may prefer not to receive information of this nature, and we should presume that existing tenets of genetic counselling, such as non-directiveness, should be respected in NICU genomic testing. As a minimum, pretest counselling should address the range of information that would be relevant to parents’ and doctors’ decisions about treatment, and should include the possibility of identifying variants of unknown significance and incidental findings. Where there is advance warning (eg, following detection of serious abnormalities on antenatal ultrasound), it may be preferable to perform such tests as a part of prenatal care. Counselling could then be undertaken prior to birth, and information would readily be available to treating doctors if a newborn then required intensive care.

However, facilitating informed decisions in the NICU will not be straightforward. We do not yet know how consent processes (particularly in the setting of parents of a critically ill infant) will be able to deal with the complexity of information and the full range of possible results of genomic testing. We do know that, if adhering to current models, it is likely to be complex and time-consuming. Genomic testing may, thus, require reassessment and revision of traditional models of informed consent.^24^

Second, given the large number of incidental and uncertain findings from genomic testing, it will be important to stratify results. Test results might be grouped into different categories based on the likelihood of them relating to the child's current or imminent clinical presentation; while incidental findings could be grouped based on clinical validity and expected utility to parents; to the extent that these things can be known.^15^ There remains considerable debate about whether some serious conditions identified incidentally on genomic testing in children should be reported.^16^ ^25^ The general approach taken to incidental findings should be the same for infants as for other children who cannot consent to testing. However, as noted above, for some critically ill infants (where life-sustaining treatment is already considered ethically optional), information about illness or function in later life that would be regarded as ‘incidental’ in other children may be relevant to parental decision-making and, hence, ‘actionable’. A decision to reveal or to filter out particular incidental findings should, in principle, be guided by what parents consider important for their child's best interests. For example, doctors might ask parents what information would change their mind about continuing or discontinuing treatment. In practice, this may be extremely challenging for parents to determine in advance.

The largest ethical dilemmas from genomic testing in the NICU will be determining whether, when and how genomic testing results should influence treatment decision-making in intensive care. To this end, a third strategy for genomic testing will be to use existing ethical frameworks for clinical decision-making in the NICU, particularly in the face of uncertainty. For example, the question of whether to perform surgery in case 2 arguably turns on an assessment of the infant's best interests, as well as on the interests and wishes of the infant's parents.^2^ ^26^ Given the high rate of morbidity following surgery, it would usually be regarded as appropriate to limit treatment and not proceed with surgery if there was agreement between the clinical team and the infant's parents. Cranial ultrasound evidence of severe intraventricular haemorrhage (IVH) would often be taken as supporting such a decision. It would, therefore, be reasonable to provide the infant's parents with genomic test results that might indicate risk of long-term problems at least of a similar severity to severe IVH.

In case 3, there might be concerns about genomic testing leading to discrimination if genetic information were used to rule out surgery. However, a number of changes revealed on genomic testing might have relevance for the likely success of transplantation or the rate of complications, factors relevant to both the infant's best interests and considerations of available health resources within a public healthcare system.^27^ ^28^ To this end, while the future implications of genomic information are important, their present utility should also be considered; particularly when it is likely to influence an imminent clinical decision.

## Conclusions

The genomic era is coming to the NICU as to other areas of medicine, and will play a role in neonatal treatment decisions. The scale of the information generated by this technology will both inform and cloud clinical decision-making. Just as with other new tests adopted in the NICU (eg, MRI), clinicians will need to treat results of genomic testing with caution, and have a critical eye to the evidence base for predictions. Parents will need to be counselled, ideally under the care of a clinical genetics service, about the uncertain and perhaps changing nature of some results.^29^ Bioinformatic analysis of genome testing is currently the rate-limiting step in providing a timely result. Additionally, much of the information needed to use genomic tests for critical care decisions is not currently available. This highlights the need to collect data that efficiently links genome testing results and neonatal long-term outcome. One important practical question (beyond the scope of this paper) relates to decisions about which test to order (eg, NGS panels, WES, WGS) and the trade-off between information return, processing time and cost. Again, the support of a clinical genetics service will be crucial for practitioners in the NICU. Genomic testing in critically ill infants is bringing to the fore difficult ethical questions about the thresholds for providing or limiting treatment, and about the role of parents in decisions. Neonatologists and parents will continue to grapple with those problems regardless of the technologies used to predict outcome in intensive care. Although the genomic era will not provide all of the answers, we believe the rapidly advancing use of genomic medicine will have long-lasting impacts on neonatal care.
